# Stereotactic Radiosurgery for Multiple Brain Metastases: Two Cases of Preserved Quality of Life

**DOI:** 10.7759/cureus.1995

**Published:** 2017-12-28

**Authors:** Anthony Pham, Becky Lee, Eric L Chang

**Affiliations:** 1 Department of Radiation Oncology, Keck School of Medicine of the University of Southern California, Los Angeles, CA; 2 David Geffen School of Medicine, UCLA

**Keywords:** brain metastases, stereotactic radiosurgery, radiation oncology, survivorship, quality of life

## Abstract

Brain metastases are the most common intracranial tumors in the adult population and have been historically treated with whole brain radiation therapy (WBRT). However, as medical advances improve life expectancy, stereotactic radiosurgery (SRS) has replaced WBRT as the standard of care for limited (one to three) brain metastases due to the relative sparing of neurocognitive function (NCF) and therefore quality of life (QoL). The use of SRS has been less documented in the case of multiple (four or more) brain metastases, with literature limited to non-randomized studies showing comparable survival and local control. In this series, we detail the case of two individuals who received SRS at our institution for multiple brain metastases and demonstrated remarkable response. The first patient is a 78-year-old woman who received Gamma Knife (GK) treatment to 17 lesions at our institution. This patient responded very well to treatment and maintains an excellent quality of life, with no deficits on serial neurological examination as she continues to travel and drive for ridesharing businesses. The second patient is an active 44-year-old woman who received SRS to 24 lesions at our institution. The patient has now been free of intracranial failures for two years and continues fulfilling her love for travel and long-distance biking. SRS is emerging as an acceptable alternative to WBRT in treating multiple brain metastases due to its preservation of NCF. Because omission of WBRT may lead to increased probability of distant brain metastasis failure, it is critical to follow these patients closely with frequent neuroimaging. In the event of a failure, it is also possible to use SRS salvage therapy with good response. Some patients who receive SRS alone demonstrate exceptional outcomes with excellent QoL, and it is possible that certain prognostication factors such as performance status, tumor histology, and tumor volume may play a role in identifying these patients. The decision to treat a patient with SRS alone for multiple brain metastases should be made carefully with consideration of systemic therapeutic options, overall prognosis, and the patient’s goals of care, with adherence to a careful follow-up plan by the physician and patient.

## Introduction

Brain metastases are the most common intracranial tumors in adults, occurring in up to 20-40% of patients diagnosed with cancer [1-2]. Historically, whole brain radiation therapy (WBRT) has been the mainstay of treatment for metastatic lesions in the brain, but it is associated with deleterious effects on neurocognitive function (NCF) and quality of life (QoL). Stereotactic radiosurgery (SRS) has emerged as a means to treat brain metastasis in a conformal manner, yielding similar overall survival as WBRT while minimizing the dose to nearby normal brain. Based on the results of phase III randomized trials that showed a decline in NCF and QoL with the addition of WBRT to SRS and/or surgery, SRS alone is now the new standard of care for the treatment of patients with limited (one to three) brain metastases [3].

To date, WBRT remains the standard approach for treatment of patients with multiple (more than four) brain metastases, as there are no completed randomized controlled trials (RCTs) to support aggressive local treatment beyond WBRT. However, non-randomized studies evaluating SRS alone in patients with four or more brain metastases have shown comparable overall survival (OS) to WBRT, with some studies achieving good local control even in patients with 10 or more lesions. This suggests SRS alone may be appropriate in these patients [4-6]. Therefore, SRS to multiple lesions in a single session has evolved to become a reasonable alternative to WBRT for patients with four or more brain metastases with good overall prognosis, especially as improvements in delivery and planning make treatment feasible. Given that some patients may live long enough to be impacted by late cognitive side effects of WBRT, we pursue a strategy of SRS alone in both the upfront and salvage setting at our institution when technically feasible and clinically appropriate. In this case series, we report the outcomes of select exceptional cases.

## Case presentation

Patient #1

A 78-year-old female first presented to our department in 2014 with BRAF mutation-positive melanoma metastatic to the brain, left adrenal gland, and spleen. Prior to this, she was in excellent health managing her household pets until she noticed an enlarging mass in the right neck behind her ear. After she hit her head on the bus she was driving, she was incidentally found to have scalp lesions as well. Both the mass and lesions were biopsied and the patient was found to have metastatic melanoma. During workup for a clinical trial, she underwent a positron emission tomography-computed tomography (PET-CT) scan in November 2013, which revealed hypermetabolic areas in the adrenal gland and spleen, in addition to bilateral scalp and neck. A magnetic resonance imaging (MRI) of the brain the same month revealed two small enhancing metastatic lesions. On the day of treatment initiation, she was found to have eight lesions and was thus treated with Gamma Knife (GK) in 2013 by the outside institution, in addition to three cycles of ipilimumab.

**Figure 1:**
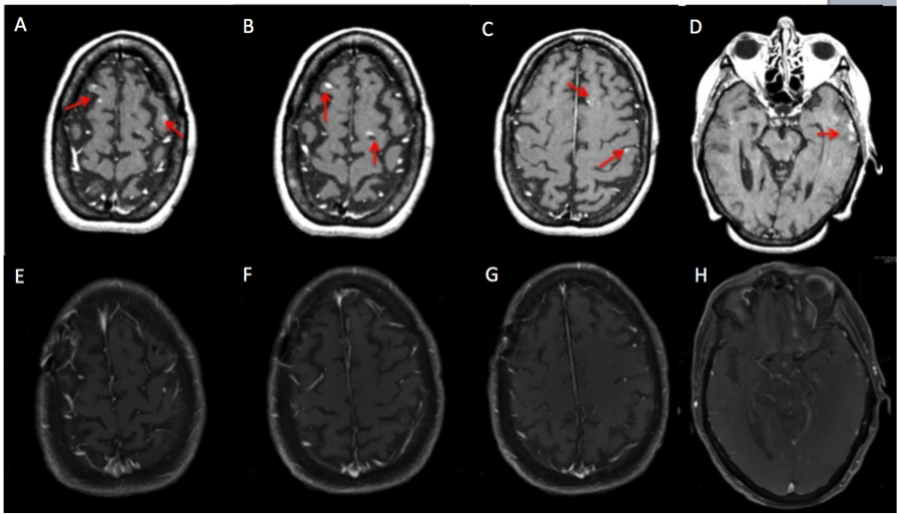
Patient PG presented with 17 lesions at the time of treatment planning. MRI of the brain including A) a right frontal lesion and a left frontal lesion, B) a right anterior frontal lesion and left posterior frontal lesion, C) a left frontal lesion and a left parietal lesion, and D) left temporal cluster. All these lesions were treated with 18 Gy to various isodose lines in one to three shots. On the most recent MRI of the brain on November 2017 (E-H), all treated lesions have disappeared and there were no new lesions concerning for metastatic disease. MRI - magnetic resonance imaging.

On interim scanning in 2014, she was found to have at least five new lesions. She was motivated to pursue further treatment with GK. On the day of her GK procedure in April 2014, imaging and evaluation by our service showed 16 lesions in the brain (Figure 1). Since she lived four hours away from our center and WBRT would be logistically difficult for the patient, the decision was made to proceed with GK. All 16 lesions were contoured and prescribed a highly conformal treatment plan receiving 18 Gy to various isodose lines in one to three shots. The patient tolerated the treatment well, but subsequent imaging in 2015 showed a right frontal lobe lesion concerning for disease progression. The patient therefore underwent a right frontal craniotomy and received postoperative GK to the lesion two months later. Since then, the patient has been on pembrolizumab maintenance treatment.

At the last follow-up in August 2017, the patient was asymptomatic and in good spirits, having recently traveled across the country and on a cruise trip to Alaska. Other than vitiligo from pembrolizumab treatment, the patient had tolerated treatment well and MRI demonstrated stable lesions. Her performance status was excellent and she had a normal neurological exam with no deficits on mini-mental status exam (MMSE). She continues to drive for multiple ridesharing companies, with aspirations for a commercial license to drive buses. The patient also cares for chickens, dogs, and a parrot on her home property, which requires performing tasks such as carrying a 50-pound bag of feed.

Patient #2

A 44-year-old previously healthy, very active female presented to the ER with a seizure while bike riding in 2013, and she was subsequently found to have metastatic BRAF-mutated melanoma to the brain and lungs. She underwent resection of her dominant left frontal lobe metastasis, followed by SRS to the resection cavity as well as four other lesions. Afterwards, she was started on ipilimumab and was asymptomatic neurologically, continuing to work regularly and bike 100 to 150 miles a week. However, later that year, she developed two more metastases that were then treated with GK, and her systemic therapy was switched to trametinib.

Unfortunately, in 2014, surveillance imaging showed multiple recurrent asymptomatic brain metastases. She also underwent lobectomy of her dominant lung lesion in August 2014 and received an interim scan while she was in the hospital and was found to have new brain metastases. During treatment, which occurred while she was still hospitalized in September 2014, nine lesions were identified. These lesions were contoured and prescribed a highly conformal treatment plan, receiving 20 Gy to various isodose lines, with two brainstem lesions receiving 18 Gy. By the end of the year, she was successfully treated for a total of 24 brain metastases, and she was switched to pembrolizumab with good response. In April 2015, she became increasingly symptomatic and was noted to have increasing edema surrounding her right posterior frontal lobe lesion, which was treated five months prior and required high dose steroids and eventually neurosurgical resection. A pathology report at this time demonstrated persistent/residual disease.

**Figure 2:**
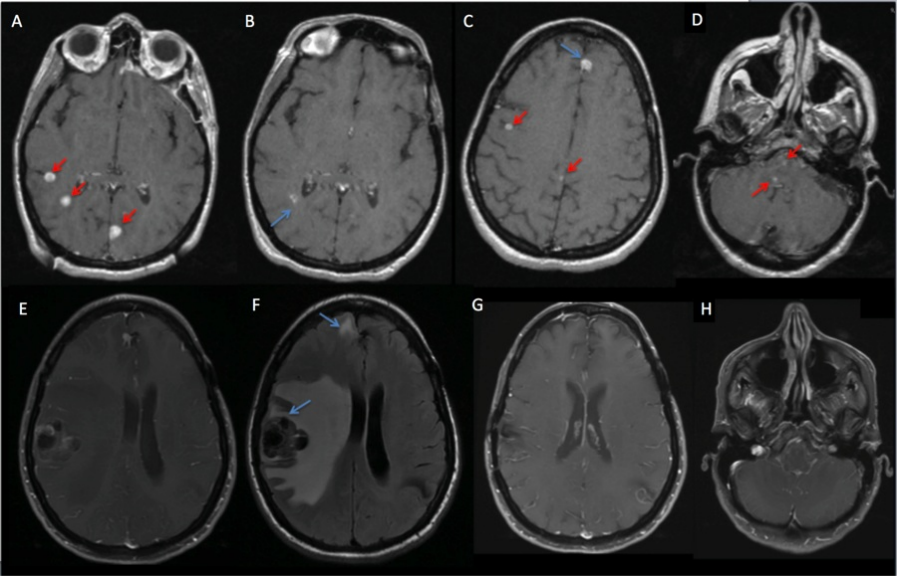
Patient MK - MRI of the brain. Patient MK initially presented in 2013 with one resection cavity and A) three untreated lesions. On her September 2014 GK MRI, her treated lesions were stable (B, blue arrow)—including a lesion treated in January 2014 (C, blue arrow); however, she presented with nine lesions, four of which are shown here (C, D, red arrows), which were treated with 20 Gy to various isodose lines, though two brainstem lesions were treated with a lower dose of 18 Gy to minimize the risk of brainstem injury (D, red arrows). In March 2015, she had persistent enhancement of a lesion (E) with surrounding edema (F), which underwent surgical resection and demonstrated persistent disease. On the most recent scan, all lesions are resolved or stable (G, H). MRI - magnetic resonance imaging, GK - Gamma Knife.

Since then, she has had only one further intracranial recurrence, which was treated with GK in the beginning of 2016. She achieved complete response on immunotherapy, which was discontinued during the summer of 2016 due to therapy-related pancreatitis and arthritis. In sum, she was treated for 37 lesions over nine GK sessions in the span of two years (Figure 2). During her last follow-up in 2017, the patient had no evidence of disease and has been free of intracranial failure for the past two years. She continues to lead a very active life, having recently returned from a vacation in Europe, and she maintains an active lifestyle biking and doing yoga.

## Discussion

Complete omission of WBRT through the use of SRS is emerging as an attractive treatment approach compared with WBRT. Technologic advances in treatment delivery and treatment planning allowing for shorter treatment times have facilitated the use of SRS alone as the primary treatment for patients with more than four lesions. The predominant justification for giving SRS for multiple brain metastases is to preserve NCF by omitting WBRT, since approximately 50% of patients will not develop distant failure despite being treated with SRS alone. Yamamoto, et al. conducted a prospective study of SRS alone in 1,194 patients with one to 10 brain metastases and found no difference in survival or treatment-related adverse events for patients with two to four and five to 10 lesions [5]. Even patients with 10 or more brain metastases treated with SRS alone may have similar oncologic and toxicity outcomes to similar patients with fewer brain metastases. A similar analysis from our institution found that there was no difference in overall survival between patients with two to four and five to 10 lesions on multivariable analysis. Factors such as a cumulative tumor volume, Karnofsky Performance Status, and histology were better potential predictors of long-term survival on multivariable analysis rather than number of lesions alone. This can be used to identify the patients who would benefit the most from SRS alone by being spared the adverse impact on NCF, with restoration to their functional baseline after treatment [4]. Hopefully, the newly opened randomized phase III trials from the Netherlands and MD Anderson investigating the NCF in patients with four or more brain metastases treated with WBRT versus stereotactic radiosurgery alone will identify patients with multiple brain metastases who can have exceptional outcomes with aggressive local intervention.

Even with the excellent outcomes of patients with good performance status and controlled extracranial disease following SRS alone for brain metastases, there is an increased risk of distant brain metastasis failure with the omission of WBRT. Nevertheless, patients who have regional failure after SRS can undergo effective salvage SRS with low morbidity and may have similar outcomes to those who experience first brain metastases with similar performance status [7-9]. Several studies have reported the efficacy of SRS after prior radiation and found similar prognostic factors to those reported in these prior studies, including younger age, longer interval from initial radiation therapy to salvage SRS, total tumor volume, and controlled extracranial disease. Aggressive local intervention should be pursued for patients with brain metastases and good performance status for whom salvage SRS is technically feasible and clinically appropriate.

## Conclusions

There is a select cohort of patients with multiple brain metastases that have exceptional outcomes. In addition to the number of brain metastases, other factors such as performance status, tumor histology, and tumor volume may play a larger role in prognostication. However, a strategy of SRS alone is dependent on the willingness of the patients and their physicians to adhere to a schedule of close monitoring that includes surveillance with high-quality MRI and a multidisciplinary team that is willing and able to perform salvage therapy when indicated. Ultimately, the decision to treat a patient with multiple brain metastases with SRS alone should be a personalized decision between the patient  and his/her care team, taking into account systemic therapeutic options, overall prognosis, and the patient’s goals of care. With continued advances in radiosurgery, we hope that these two exceptional cases will be the norm for all patients with brain metastases.
